# Right wing authoritarianism is associated with race bias in face detection

**DOI:** 10.1371/journal.pone.0179894

**Published:** 2017-07-10

**Authors:** Amélie Bret, Brice Beffara, Jessica McFadyen, Martial Mermillod

**Affiliations:** 1 Univ. Grenoble Alpes, LPNC, Grenoble, France; 2 CNRS, LPNC UMR 5105, Grenoble, France; 3 IPSY, Université Catholique de Louvain, Louvain-la-Neuve, Belgium; 4 The Queensland Brain Institute, The University of Queensland, St Lucia QLD Australia; 5 Institut Universitaire de France, Paris, France; Centre national de la recherche scientifique, FRANCE

## Abstract

Racial discrimination can be observed in a wide range of psychological processes, including even the earliest phases of face detection. It remains unclear, however, whether racially-biased low-level face processing is influenced by ideologies, such as right wing authoritarianism or social dominance orientation. In the current study, we hypothesized that socio-political ideologies such as these can substantially predict perceptive racial bias during early perception. To test this hypothesis, 67 participants detected faces within arrays of neutral objects. The faces were either Caucasian (in-group) or North African (out-group) and either had a neutral or angry expression. Results showed that participants with higher self-reported right-wing authoritarianism were more likely to show slower response times for detecting out- vs. in-groups faces. We interpreted our results according to the Dual Process Motivational Model and suggest that socio-political ideologies may foster early racial bias via attentional disengagement.

## Introduction

North and Sub Saharan African immigrants and children of immigrants are among the most discriminated individuals in Western Europe [1]. Discrimination arises from prejudices, defined as positive and negative attitudes shaped by individual experience. Right Wing Authoritarianism (RWA) and Social Dominance Orientation (SDO) are substantial predictors of inter-group prejudices. RWA describes the tendency for an individual to submit to authorities, exert authoritarian aggression, and adhere to social conventions. SDO describes the extent to which an individual prefers social hierarchy [2]. Although these two personality traits are positively correlated, they are underpinned by distinct mechanisms [3]. RWA is mainly linked to social conformity and threat perception, whereas SDO centers on competition and hierarchy. There is consistent evidence to support this dual process of RWA and SDO in contributing to prejudice; however, it is still not clearly understood how this dual process influences early information processing, such as face detection.

In humans, the face is probably the most meaningful tool during social interaction [4]. Racial biases have been robustly reported in face detection but the effects may depend on context, culture and task [5]. The dependency of racial bias upon these factors is not surprising given that high-level conceptual or social information are known to modulate low-level mechanisms of visual perception [6,7,8]. Affective valence modulates object identification during visual perception [9]but personality traits, ideologies, motivation, and social context also play a large part in face processing and categorization [10,11,12,13]. It is therefore likely that socio-political ideologies explain early differences in the perception of in-group and out-group members. One hypothesis for this is that individuals with greater RWA and SDO scores require more time to detect out-group faces because they grant less attention to other-race individuals [14]. On the other hand, a second hypothesis is that these individuals are quicker to detect out-group faces because they are more threatening for the in-group and should therefore deserve priority in early perception [15].

To untangle these two competing hypotheses, we designed a visual search task where participants detected a face, either Caucasian (CA) or North-African (NA) and with either a neutral or angry expression, amongst neutral objects. For a sample of Caucasian participants, longer response times for NA vs. CA would reflect less attentional engagement toward out-group faces (first hypothesis). On the other hand, shorter response times for NA vs. CA would reflect increased attentional capture by out-group faces (second hypothesis). Furthermore, we predicted that perceived threat (manipulated by either a neutral or angry expression) would modulate the response times of high RWA and SDO participants compared to low RWA and SDO participants, possibly more so for out- vs. in-group faces.

## Method & material

### Participants

Sixty-seven Caucasian volunteers participants from Grenoble Alpes University (59 females), 18 to 53 years of age (*M* = 20.37, *SD* = 4.49), completed the experiment for course credit. Participants were informed of the experiment procedure and then completed an oral and written consent form before they started the experiment. They were free to leave the experiment at any time without any consequences. A complete debriefing was given at the end of the experiment.

### Material

We used 210 neutral stimuli from the NAPS database for objects [16]. Face stimuli were selected from the ADFES database [17], resulting in ten images selected for each stimulus category (NA neutral, NA angry, CA neutral, CA angry), composed equally of male and female faces. The ethnic identification of these stimuli has previously been validated, such that participants identified the Caucasian individuals as more native European (M = 5.19, SD = .79) compared to North-African individuals (M = 2.62, SD = .80)[17]. The stimuli were displayed on a 19” screen at a resolution of 1280 x 1024 pixels. Participants were approximately seated at 70cm from the screen.

We used a Right-Wing Authoritarianism Questionnaire short version of the Altemeyer’s 30-item [18]. We administered the 10 items version (α = .833) from the French translation RWA Scale (1 = totally disagree to 7 = totally agree) developed by Haddock, Zanna, and Esses [19] and Social Dominance Orientation 10 items (α = .838) from the French translation of the 16-items version [20,21] of the SDO Scale (1 = totally disagree to 7 = totally agree).

#### Ethics statement

All stimuli and questionnaires used in our task have been approved by the ethical committee of the Catholic University of Louvain, Belgium (reference #Projet2015-38Bis). Ethical approval was not required in France for this study. All participants provided informed written consent.

### Procedure

Participants were instructed to report as fast and as accurately as possible whether a face was present in an 8 x 8 search array displayed on the computer screen. The face was either angry or neutral and either Caucasian or North African but this information was neither part of the instructions nor explicitly stated to the participants. 160 trials (80 trials with a face and 80 trials without a face) were randomly distributed. A 500ms fixation cross was displayed between each trail. A 30s break was introduced after one half of the trials were presented. The search array remained on screen until the participant’s response. After completing the experiment, participants completed the questionnaires measuring RWA and SDO.

## Results

We analyzed data from 62 of the 67 participants (exclusion criteria: 1 for < 75% accuracy, 1 Cook’s Distance response time outlier, and 3 due to technical issues). Across this sample, no significant differences in response time were found. Scores for RWA and SDO were strongly correlated (r(60) = .60, p < .001). To investigate potential predictive effects of socio-political conservatism, we performed two separate linear regressions on the difference in response time between CA and NA: one with RWA as a predictor and one with SDO as a predictor. The dependent variable was the response time difference between CA and NA faces, collapsed across emotional expression.

**Fig 1:**
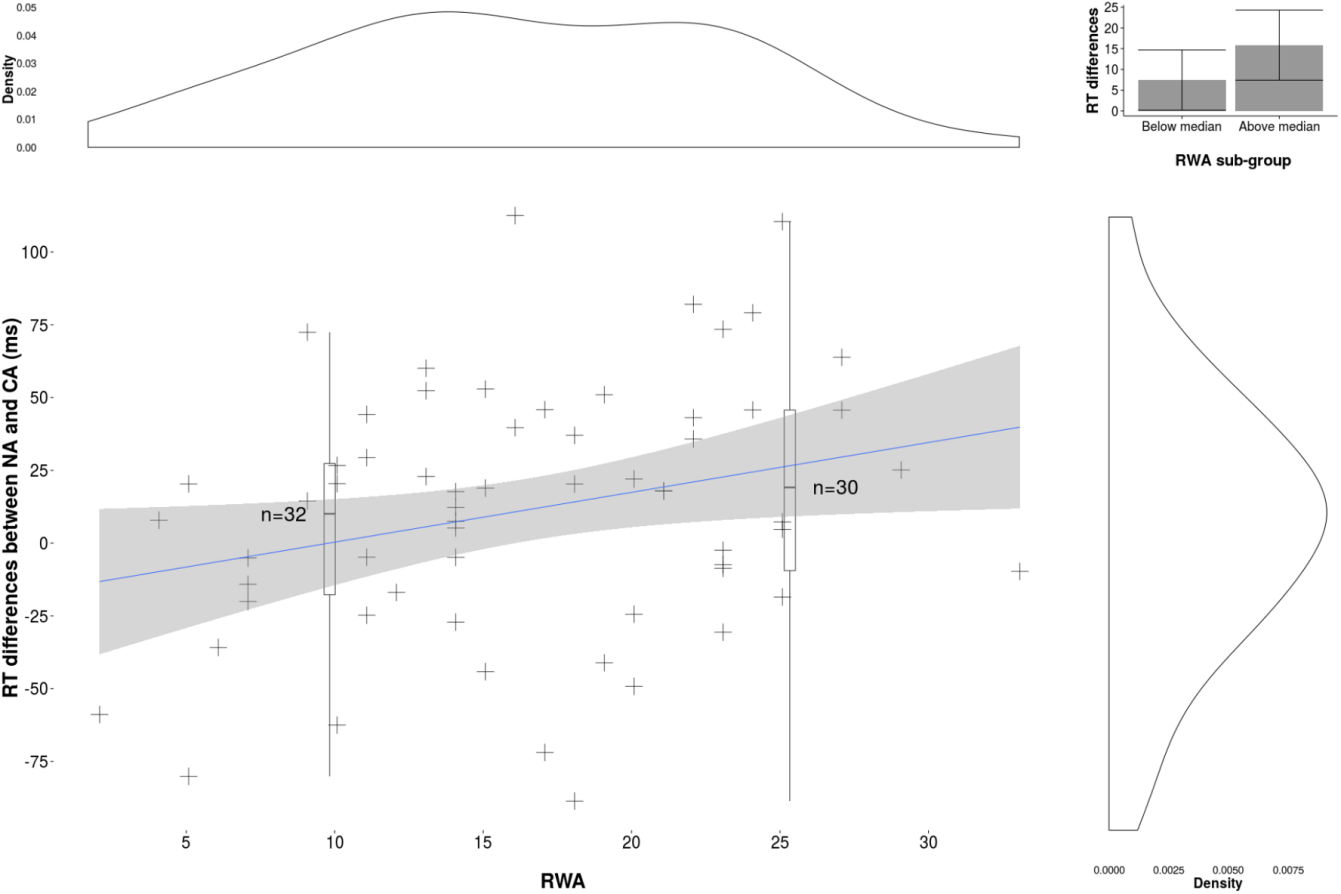
Response time differences between Caucasian and North African faces (ms). Density represents the data distribution for each variable (axis) *Note*, *N* = 62.

The RWA regression analysis revealed that RWA explained a significant amount of variance in response time differences between in- and out-group faces (F(1,61) = 4.81, Beta = 2.29 *p* < .05, 95% CI [0.17, 4.24],*R*^*2*^ = .07). Critically, participants with higher RWA scores showed slowed response times to detect NA compared to CA faces (Fig 1). We followed this up with two regression analyses on the simple effects of emotion (i.e. CA-Neutral vs. NA-Neutral, CA-Angry vs. NA-Angry) but these did not yield significant results (see Tables 1 and 2). The second SDO regression analysis also returned no significant results.

**Table 1:** Linear regressions examining the associations between RWA, SDO and response time (RWA and SDO are centered).

	CA vs. NA			CA-Neutral vs. NA-Neutral			CA-Angry vs. NA-Angry		
	*β*	*t*	*p*	*β*	*t*	*p*	*β*	*t*	*p*
**RWA**	-2.293	-2.218	.04	-1.563	-1.881	.03	-.730	-1.157	NS
**SDO**	0.021	-1.25	NS	0.024	0.61	NS	0.00	-0.015	NS

**Table 2:** Descriptive statistics (Mean and Standard deviation).

Type of faces	*M*	*SD*
CA	521.84	83.27
CA neg	522.13	76.83
NA	533.35	79.07
NA neg	524.23	78.42

## Discussion

This study was designed to investigate the association between socio-political ideologies and racial biases in a face detection task. We found evidence to support such an association: RWA predicted longer response times for detecting NA vs. CA faces. To our knowledge, this study is the first to have investigated i) perception differences between CA and NA faces in a visual search task, and ii) the association between socio-political ideologies and these perception differences. Critically, we discovered that that the slower detection of NA faces by participants with higher RWA was independent of the face’s emotional expression. This effect could be explained by a lack of attention allocated to out-group members, as opposed to the perceived threat hypothesis. Our results are surprising considering the existing literature, which predicted that SDO, more than RWA, should be associated with a difference in this direction. Indeed, as mentioned before, RWA should be associated with perception of threat in out-group members and SDO should predict lower motivation to process out-group faces [3]. However, we did not find significant results concerning SDO and thus our results do not support an association between this socio-political ideology and low-level (i.e. perceptual) correlates of prejudice/discrimination.

Performance in the visual search task demonstrated that individuals with higher prejudice against out-group members (i.e. higher RWA) were slower to detect out-group faces. This could relate to an intrinsic motivation to assign less attention to out-group members, perhaps because they are perceived as less relevant. However, the possibility remains that threat perception may emerge in a delayed second processing step [22]. Future studies should investigate different moderators (such as side attentional measures) so as to determine whether attentional disengagement could lead to threat perception and therefore reconcile our results with the dual-process motivational model [3].

Of further interest, our results also illustrated an early threat perception process provoked by NA faces on higher RWA participants. Indeed, previous research highlighted that avoidance was a central personality style influencing out-group perception ([23,24,25]. As a consequence, attentional disengagement from NA faces could be the strategy by which higher RWA participants manage (i.e. avoid) threat. The avoidance hypothesis probably constitutes the richest framework of interpretation. More specifically, the defensive avoidance effect allows to bring the two previous possible mechanisms, namely, threat and disengagement, together. Defensive avoidance is the way by which individuals selectively screen out decision-contrary information [26]. Although this mechanism can be observed in the large context of cognitive dissonance, [27]showed that this strategy was common in highly authoritarian individuals in situations of threat, as compared to low authoritarian individuals. Avoiding information is part of a coping mechanism allowing to reduce anxiety, in particular in the case of extremist behavior related to prejudice or obedience to authority [28]. Indeed, disengaging from information potentially related to threatening events acts as a buffer against anxious states caused by the perceived threatening world, event in absence of an objective threatening event[29]. In the context of the present study, high RWA participants might disengage (i.e. perform defensive avoidance) from NA faces not in spite of but because of the threat potentially related to these faces.

Finally, this effect is to be interpreted as a contrast of NA compared to CA faces perception. Indeed, our dependent variable measures a bias between in- and out-group faces perception. Hence, preference for in-group faces is likely also involved in our observed response latency differences, promoting approach behavior. This positive bias for in-group faces could therefore underlie the gap between early automatic attention orienting toward CA versus NA faces ([30,31,32,33]

Our results must be interpreted by taking into consideration potential potentially important methodological point. We know that self-reported measures of political ideologies can be affected by previous experimental tasks performed by participants [34,35]. Even if implicit tasks, such as ours, are less likely to influence participants’ answers to questionnaires [36,37], it is important to consider the question of task order. The face detection task and the questionnaires could possibly influence each other depending of the order of presentation (e.g., experimental asking bias, priming effects). In this case, participants always completed the questionnaire *after* the face detection task and thus there may be an influential effect of having just seen expressive CA and NA faces. A design such as this raises important methodological questions; namely, is there a better (i.e. less biased) order of presentation and is there a need to consider both orders in a counterbalanced designed? As a consequence, because we cannot answer this question with our own design, there is a strong need for future studies to explore the influence of answering the questionnaires before (versus after) the face detection task.

To summarize, we found that RWA, a sub-component of right-wing socio-political ideologies, predicted differences in different-race face perception. We concluded that this effect was likely driven by decreased attentional engagement toward out-group members. It is important to note that RWA explained only 7% of face detection variance. This means that other predictors have to be considered alongside this top-down mechanism. Further studies are required to understand the underlying mechanisms and potential interactions between situational and dispositional factors.

## References

[1] International Migration Outlook 2014. Paris: OECD Publishing; 2017.

[2] Duckitt J, Sibley C. Right wing authoritarianism, social dominance orientation and the dimensions of generalized prejudice. European Journal of Personality. 2007;21(2):113–130.

[3] Duckitt J, Sibley C. Personality, Ideology, Prejudice, and Politics: A Dual-Process Motivational Model. Journal of Personality. 2010;78(6):1861–1894. doi: 10.1111/j.1467-6494.2010.00672.x 21039534 10.1111/j.1467-6494.2010.00672.x

[4] Jack R, Schyns P. The Human Face as a Dynamic Tool for Social Communication. Current Biology. 2015;25(14):R621–R634. doi: 10.1016/j.cub.2015.05.052 26196493 10.1016/j.cub.2015.05.052

[5] Sun G, Song L, Bentin S, Yang Y, Zhao L. Visual search for faces by race: A cross-race study. Vision Research. 2013;89:39–46. doi: 10.1016/j.visres.2013.07.001 23867566 10.1016/j.visres.2013.07.001

[6] Beffara B, Wicker B, Vermeulen N, Ouellet M, Bret A, Molina M et al Reduction of interference effect by low spatial frequency information priming in an emotional Stroop task. Journal of Vision. 2015;15(6):16 doi: 10.1167/15.6.16 26024463 10.1167/15.6.16

[7] Kveraga K, Ghuman A, Bar M. Top-down predictions in the cognitive brain. Brain and Cognition. 2007;65(2):145–168. doi: 10.1016/j.bandc.2007.06.007 17923222 10.1016/j.bandc.2007.06.007 PMC2099308

[8] Kever A, Grynberg D, Eeckhout C, Mermillod M, Fantini C, Vermeulen N. The body language: The spontaneous influence of congruent bodily arousal on the awareness of emotional words. Journal of Experimental Psychology: Human Perception and Performance. 2015;41(3):582–589. doi: 10.1037/xhp0000055 25915069 10.1037/xhp0000055

[9] Barrett L, Bar M. See it with feeling: affective predictions during object perception. Philosophical Transactions of the Royal Society B: Biological Sciences. 2009;364(1521):1325–1334. 10.1098/rstb.2008.0312 PMC2666711 19528014

[10] Beffara B, Ouellet M, Vermeulen N, Basu A, Morisseau T, Mermillod M. Enhanced embodied response following ambiguous emotional processing. Cognitive Processing. 2012;13(S1):103–106. 10.1007/s10339-012-0468-6 22802035

[11] Calder A, Ewbank M, Passamonti L. Personality influences the neural responses to viewing facial expressions of emotion. Philosophical Transactions of the Royal Society B: Biological Sciences. 2011;366(1571):1684–1701. 10.1098/rstb.2010.0362 PMC3130379 21536554

[12] Krosch A, Berntsen L, Amodio D, Jost J, Van Bavel J. On the ideology of hypodescent: Political conservatism predicts categorization of racially ambiguous faces as Black. Journal of Experimental Social Psychology. 2013;49(6):1196–1203.

[13] Villepoux A, Vermeulen N, Niedenthal P, Mermillod M. Evidence of fast and automatic gender bias in affective priming. Journal of Cognitive Psychology. 2015;27(3):301–309.

[14] Haslam N, Loughnan S. Dehumanization and Infrahumanization. Annual Review of Psychology. 2014;65(1):399–423. 10.1146/annurev-psych-010213-115045 23808915

[15] Trawalter S, Todd A, Baird A, Richeson J. Attending to threat: Race-based patterns of selective attention. Journal of Experimental Social Psychology. 2008;44(5):1322–1327. doi: 10.1016/j.jesp.2008.03.006 19727428 10.1016/j.jesp.2008.03.006 PMC2633407

[16] Marchewka A, Żurawski Ł, Jednoróg K, Grabowska A. The Nencki Affective Picture System (NAPS): Introduction to a novel, standardized, wide-range, high-quality, realistic picture database. Behavior Research Methods. 2013;46(2):596–610. 10.3758/s13428-013-0379-1 PMC4030128 23996831

[17] van der Schalk J, Hawk S, Fischer A, Doosje B. Moving faces, looking places: Validation of the Amsterdam Dynamic Facial Expression Set (ADFES). Emotion. 2011;11(4):907–920. doi: 10.1037/a0023853 21859206 10.1037/a0023853

[18] Enemies of freedom: understanding right-wing authoritarianism. Choice Reviews Online. 1989;26(07):26-3934-26-3934.

[19] Haddock G, Zanna M, Esses V. Assessing the structure of prejudicial attitudes: The case of attitudes toward homosexuals. Journal of Personality and Social Psychology. 1993;65(6):1105–1118.

[20] Duarte S, Dambrun M, Guimond S. DUARTE S., DAMBRUN M., et GUIMOND S. Social dominance and legitimizing myths: Validation of a French form of the social dominance orientation scale. Revue Internationale de Psychologie Sociale, 2004, vol. 4, p. 97–126. Revue Internationale de Psychologie Sociale. 2004;4:97–126.

[21] Pratto F, Sidanius J, Stallworth L, Malle B. Social dominance orientation: A personality variable predicting social and political attitudes. Journal of Personality and Social Psychology. 1994;67(4):741–763.

[22] Pereira C, Vala J, Costa-Lopes R. From prejudice to discrimination: The legitimizing role of perceived threat in discrimination against immigrants. European Journal of Social Psychology. 2010;40(7):1231–1250. [25]

[23] Terrizzi J, Shook N, Ventis W. Disgust: A predictor of social conservatism and prejudicial attitudes toward homosexuals. Personality and Individual Differences. 2010;49(6):587–592.

[24] Choma B, Hodson G, Costello K. Intergroup disgust sensitivity as a predictor of islamophobia: The modulating effect of fear. Journal of Experimental Social Psychology. 2012;48(2):499–506.

[25] Hodson G, Costello K, Macinnis C. Is intergroup contact beneficial among intolerant people. Advances in intergroup contact. 2013;:49–80.

[26] Garrett R, Carnahan D, Lynch E. A Turn Toward Avoidance? Selective Exposure to Online Political Information, 2004–2008. Political Behavior. 2011;35(1):113–134.

[27] Lavine H, Lodge M, Freitas K. Threat, Authoritarianism, and Selective Exposure to Information. Political Psychology. 2005;26(2):219–244.

[28] Mermillod M, Marchand V, Lepage J, Begue L, Dambrun M. Destructive Obedience Without Pressure. Social Psychology. 2015;46(6):345–351.

[29] Hinckley R. Authoritarianism, Selective Exposure, and Political Intolerance. SSRN Electronic Journal.

[30] Dickter C, Bartholow B. Racial ingroup and outgroup attention biases revealed by event-related brain potentials. Social Cognitive and Affective Neuroscience. 2007;2(3):189–198. doi: 10.1093/scan/nsm012 18985140 10.1093/scan/nsm012 PMC2569810

[31] Bernstein M, Young S, Hugenberg K. The Cross-Category Effect. Psychological Science. 2007;18(8):706–712. doi: 10.1111/j.1467-9280.2007.01964.x 17680942 10.1111/j.1467-9280.2007.01964.x

[32] Van Bavel J, Packer D, Cunningham W. The Neural Substrates of In-Group Bias: A Functional Magnetic Resonance Imaging Investigation. Psychological Science. 2008;19(11):1131–1139. doi: 10.1111/j.1467-9280.2008.02214.x 19076485 10.1111/j.1467-9280.2008.02214.x

[33] Van Bavel J, Packer D, Cunningham W. Modulation of the Fusiform Face Area following Minimal Exposure to Motivationally Relevant Faces: Evidence of In-group Enhancement (Not Out-group Disregard). Journal of Cognitive Neuroscience. 2011;23(11):3343–3354. doi: 10.1162/jocn_a_00016 21452952 10.1162/jocn_a_00016

[34] Huang L, Liu J. Personality and social structural implications of the situational priming of social dominance orientation. Personality and Individual Differences. 2005;38(2):267–276.

[35] Bryan C, Dweck C, Ross L, Kay A, Mislavsky N. Political mindset: Effects of schema priming on liberal-conservative political positions. Journal of Experimental Social Psychology. 2009;45(4):890–895.

[36] Thomas C, Esses V. Individual Differences in Reactions to Sexist Humor. Group Processes & Intergroup Relations. 2004;7(1):89–100.

[37] Lipp O, Derakshan N. Attentional bias to pictures of fear-relevant animals in a dot probe task. Emotion. 2005;5(3):365–369. doi: 10.1037/1528-3542.5.3.365 16187873 10.1037/1528-3542.5.3.365

